# Evaluation of Antioxidant and Antiproliferative Properties of Three *Actinidia* (*Actinidia kolomikta*, *Actinidia arguta*, *Actinidia chinensis*) Extracts *in Vitro*

**DOI:** 10.3390/ijms13055506

**Published:** 2012-05-08

**Authors:** Li-Li Zuo, Zhen-Yu Wang, Zi-Luan Fan, Shuang-Qi Tian, Jia-Ren Liu

**Affiliations:** 1 School of Food Science and Engineering, Harbin Institute of Technology, 73 HuangHe Road, NanGang District, Harbin 150090, China; E-Mails: zuolili213@163.com (L.-L.Z.); fzl_1122@163.com (Z.-L.F.); tianshuangqi2002@163.com (S.-Q.T.); 2 School of Forestry, Northeast Forestry University, 26 HeXing Road, DongLi District, Harbin 150040, China; 3 Harvard Medical School, 300 Longwood Ave., Boston, MA 02115-5737, USA; E-Mail: Jia-ren.liu@childrens.harvard.edu

**Keywords:** *Actinidia*, total phenolic content, flavonoids, antioxidant activity, antiproliferative activity

## Abstract

The total phenolic content, total flavonoid content, vitamin C content, and antioxidant activities of ethanol extracts from different kiwifruit varieties (*Actinidia kolomikta*, *Actinidia arguta*, *Actinidia chinensis*) were determined in this study. Multiple scavenging activity assays including the hydroxyl radical, O_2_^−^·radical, DPPH, and the ABTS^+^ radical scavenging activity assays were used to identify the antioxidant activities of *Actinidia* extracts. The cell viability of HepG2 and HT-29 cells was also examined in this study. The results demonstrated that the *Actinidia kolomikta* extract had a higher antioxidant activity than the other two *Actinidia* extracts. There is a positive correlation between antioxidant activity and the polyphenols and vitamin C content in all three extracts (*R*^2^ ≥ 0.712, *p* < 0.05). The *Actinidia arguta* extract had the highest inhibitory effect on HepG2 and HT-29 cell growth. These results provide new insight into the health functions of fruit and demonstrate that *Actinidia* extracts can potentially have health benefits.

## 1. Introduction

There is increasing interest regarding the use of naturally occurring antioxidants in human food and nutraceutical products to replace synthetic antioxidants [1]. Natural antioxidants are safer and healthier than synthetic antioxidants. The term “antioxidants” refers to such a group of compounds that are able to delay or inhibit the oxidation of biomolecules, and they prevent or repair the damage caused by free radicals in body cells [2]. In an organism, free radicals are an unstable species that react rapidly and destructively with biomolecules such as protein, lipids, DNA, and RNA in the human body [3]. Furthermore, polyphenol-rich foods, such as apple and grape seeds effectively diminish DNA oxidation damage by reducing the reactive oxygen species (ROS) level [4]. Accordingly, natural antioxidants play an important role in defending the human body against the damaging effects of free radicals [5,6].

Recent studies have found that extracts from natural products, such as fruits, vegetables and medicinal herbs, had a positive effect against cancer, compared to chemotherapy and hormonal treatments [7–9]. Epidemiological and laboratory studies have also shown that taking abundant fruits and vegetables in the human diet is associated with a lower risk of heart disease and cancer [10]. Fruits and vegetables contain many phytochemicals with various bioactivities including antioxidant, anti-inflammatory and anticancer activities. In a previous study, it was found that fruits provide the largest contribution of antioxidants in the human diet due to an abundance of vitamins, phenolic compounds, and carotenoids [11]. Zulueta and colleagues calculated that fruit juices (grape juice, grapefruit juice *etc.*) contributed 5–6% of the total antioxidant capacity in a Spanish diet [12]. Other medicinal plants, such as *Diospyros abyssinica*, *Pistacia lentiscus*, *Geranium sanguineum L.*, *Dracocephalum moldavica L*. have been found to have potential antioxidant activities [13,14]. *Platycodon grandiflorum* also contains strong anticancer compounds that have been found to display cytotoxicity on three human cancer cell lines [15].

The *Actinidia chinensis* plant is native to Southern China. *Actinidia kolomikta* and *Actinidia arguta* are widely consumed in the DaXingAnLing region of northeastern China. A recent study by Du *et al.* found that *Actinidia chinensis* has high antioxidant activity. The wild *Actinidia eriantha* and *A. latifolia* species have significantly higher antioxidant capacity than the cultivars of *Actinidia chinensis* and *Actinidia deliciosa* [16]. Ascorbic acid, which is a potential source of antioxidants, is different among the six genotypes of *Actinidia chinensis* [17]. Bioactivities of *Actinidia* are influenced by species and cultivars. However, the bioactivities of the three above-mentioned *Actinidia* are still unclear. Thus, the objectives of this study were to evaluate the total phenolic and total flavonoid content, vitamin C, antioxidant activity, and antiproliferative activity from an ethanol extract of each *Actinidia*, and to test how they affect human cancer cell lines.

## 2. Results and Discussion

### 2.1. The Total Phenolic, Total Flavonoids and Vitamin C Contents

**Figure 1:**
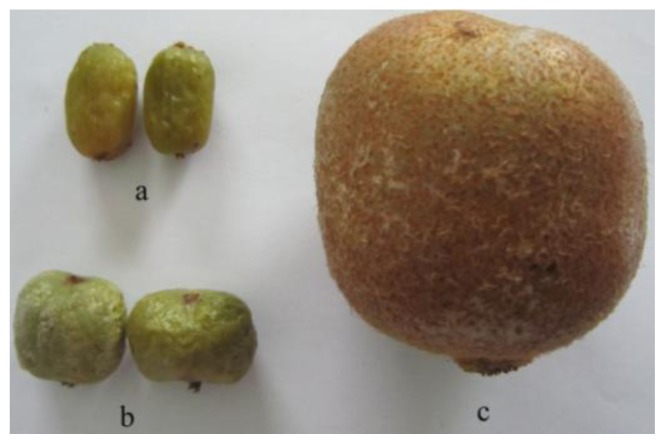
Fruits. (**a**) Actinidia kolomikta; (**b**) Actinidia Arguta; (**c**) Actinidia Chinensis.

Three different varieties, *Actinidia kolomikta*, *Actinidia arguta*, *Actinidia chinensis*, were obtained from a local market (Harbin, China) (Figure 1). The total phenolic content (TPC), total flavonoid content (TFC) and vitamin C content of *Actinidia* extracts is shown in Table 1. The results showed a high phenolic content of the ethanol extract from *Actinidia kolomikta*. In this study, the TPC of *Actinidia* ethanol extract displayed the following order: *Actinidia kolomikta* > *Actinidia arguta* > *Actinidia chinensis*. Thus, *Actinidia kolomikta* has the highest TPC and it is a potential source of antioxidants.

Flavonoids are the most common compounds and are a widely distributed group of phenolic compounds in plants. Flavonoids are usually considered as effective antioxidants. In this study, the TFC of three *Actinidia* ethanol extracts was established using an aluminum colorimetric assay. The TFC value in the three *Actinidia* extracts showed decreasing order: *Actinidia arguta* > *Actinidia kolomikta* > *Actinidia chinensis*. Studies have shown that the TFC in eight different *Actinidia* genotypes ranged from 3.01 to 91.79 mg CE/100 g FW [16]. Thus, the flavonoid content in *Actinidia* is strongly influenced by species and cultivars. In this study, the *Actinidia kolomikta* extract had the highest vitamin C content, followed by *Actinidia chinensis* and *Actinidia arguta*. The vitamin C content in Californian-grown cantaloupe and honeydew melons are 36.7 ± 1.38 mg/100 g FW and 18 ± 1.64 mg/100 g FW, respectively [18]. Thus, the vitamin C contents of *Actinidia* are generally comparable to Californian-grown melons. Therefore, based on the Californian-grown melon measurements, the *Actinidia* extracts showed high vitamin C contents.

### 2.2. Antioxidant Activity

**Figure 2:**
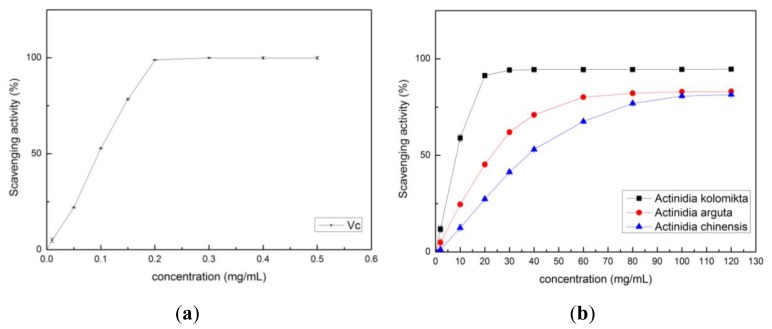
(**a**) Hydroxyl radical scavenging activity of vitamin C; (**b**) Hydroxyl radical scavenging activity of three *Actinidia* ethanol extracts.

**Figure 3:**
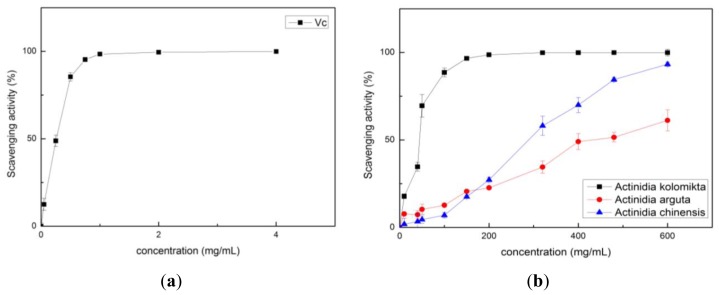
**(a)** The O_2_^−^ scavenging activity of vitamin C; (**b**) The O_2_^−^ scavenging activity of three *Actinidia* ethanol extracts.

**Figure 4:**
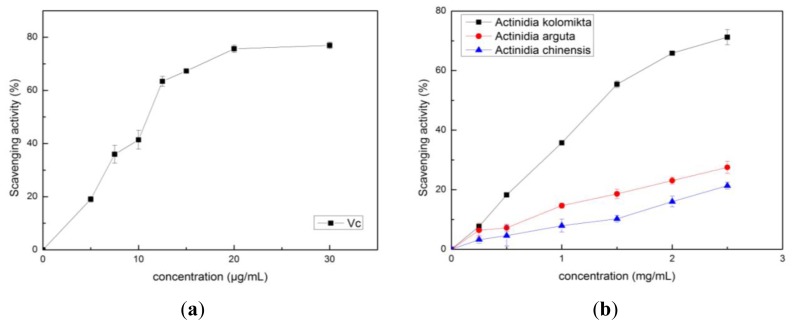
(**a**) The DPPH scavenging activity of vitamin C; (**b**) The DPPH scavenging activity of three *Actinidia* ethanol extracts.

**Figure 5:**
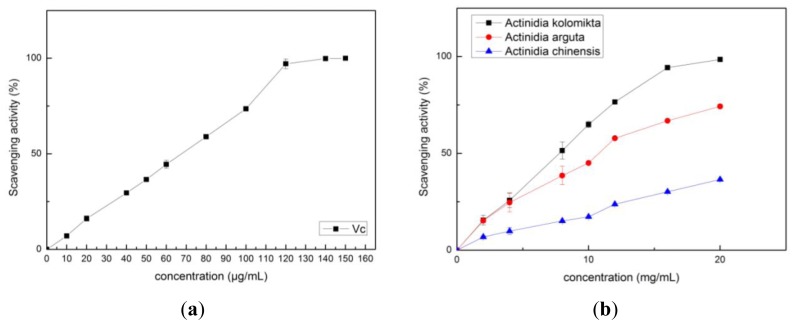
(**a**) The ABTS scavenging activity of vitamin C; (**b**) The ABTS scavenging activity of three *Actinidia* ethanol extracts.

Free radicals are known to be major factors in triggering biological damage such as DNA, RNA, protein, or lipid oxidation [19]. Most antioxidants that exist in plants have radical-scavenging capacity. The common methods of measuring the free radical scavenging activity of antioxidants are by using the hydroxyl radical, O_2_^−^ radical, DPPH, and ABTS^+^ assays. The hydroxyl radical and O_2_^−^ radical scavenging activity assays are widely used in measuring food antioxidant capacity [20]. The radical scavenging capacity of each *Actinidia* extract was measured using Equation (1) in this study, and the results are shown in Figures 2–5. The radical-scavenging capacity of *Actinidia kolomikta* at a dose of 20 mg FW/mL was 91.31%. This result was similar to 0.2 mg/mL of vitamin C (Figure 2). The free radical scavenging activity is usually expressed as percentage of hydroxyl radical or O_2_^−^ radical inhibition, and can also be expressed by the antioxidant concentration required for a 50% hydroxyl radical or O_2_^−^ radical (IC_50_). A low IC_50_ value indicates high antioxidant ability. The IC_50_ values were 6.61 ± 0.24, 21.72 ± 0.65 and 38.29 ± 0.52 mg/mL in the extracts of *Actinidia kolomikta*, *Actinidia arguta*, and *Actinidia chinensis*, respectively.

(1) $$\text{Scavenging }(\text{or inhibition})\;\text{rate }(\%)=[1-({\text{A}}_{1}-{\text{A}}_{2})/{\text{A}}_{0}]\times 100\%$$

In our study, the vitamin C and *Actinidia* extracts showed strong scavenging abilities of O_2_^−^ radicals in a dose-dependent manner (Figure 3). The *Actinidia kolomikta*, at a dose of 80 mg FW/mL, displayed the same 96.6% of radical scavenging activity as 0.75 mg/mL of vitamin C (95.3%). The IC_50_ values of O_2_^−^ scavenging activity were 22.68 ± 1.81, 241.64 ± 4.55 and 592.28 ± 8.35 mg/mL for *Actinidia kolomikta*, *Actinidia chinensis* and *Actinidia arguta*, respectively. It is known that the phenolics with hydroxyl groups possess a superoxide anion scavenging activity by their electron donation [21]. The results showed that *Actinidia* extracts demonstrated a scavenging activity of the superoxide anion.

The free radical scavenging activities of *Actinidia* extracts and vitamin C were determined by using the DPPH and ABTS^+^ assays. The results are shown in Figures 4 and 5. The IC_50_ values of DPPH were 1.35 ± 0.04, 9.89 ± 0.75 and 13.97 ± 0.42 mg/mL, and the IC_50_ values in the ABTS^+^ assay, 5.68 ± 0.20, 9.71 ± 0.39 and 45.60 ± 0.57 mg/mL for *Actinidia kolomikta*, *Actinidia arguta* and *Actinidia chinensis*, respectively. Thus, like vitamin C, each *Actinidia* extract had potent free radical scavenging activities. The principal component analysis of the antioxidant activities in each *Actinidia* extract also showed this tendency, and decreased in the following order: *Actinidia kolomikta* > *Actinidia arguta* > *Actinidia chinensis*. The findings showed that a positive correlation was found between the TPC or vitamin C and the scavenging activities (Table 2) (*p* < 0.05). In our study, vitamin C content was strongly correlated with antioxidant capacity. Our data indicate that total phenolic and vitamin C of *Actinidia* fruits were major sources of natural antioxidants. Thus, the antioxidant capacity of *Actinidia* appears to be largely influenced by the polyphenols and vitamin C content.

Our data also showed that *Actinidia* has generally high antioxidant capacity. The wild *Actinidia* species (*Actinidia kolomikta)* has stronger antioxidant activities than the *Actinidia arguta* or *Actinidia chinensis*. Thus, both polyphenols and vitamin C are major contributors to the total antioxidant capacity of *Actinidia* fruits.

### 2.3. Effect of *Actinidia* Extracts on Cancer Cell Proliferation

**Figure 6:**
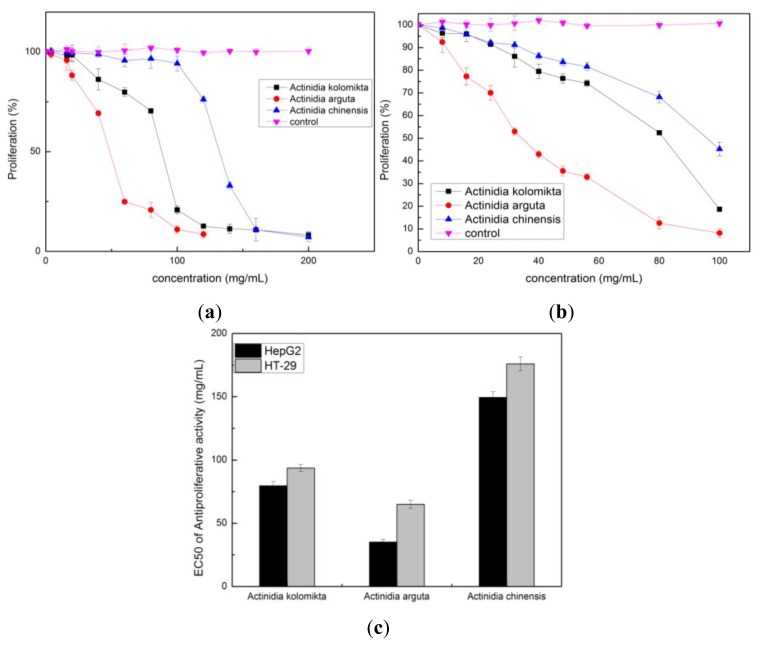
(**a**) Inhibition of HT-29 cell proliferation by the treatments of different concentrations of *Actinidia* extracts for 48 h; (**b**) Inhibition of HepG2 cell proliferation by the treatments of different concentrations of *Actinidia* extracts for 48 h; (**c**) EC_50_ values of antiproliferative activity of three *Actinidia* extracts on HepG2 and HT-29 (mean ± SD, *n* = 3).

The antiproliferative activity of each *Actinidia* extract on HT-29 and HepG2 cells was examined by MTT assay. A mitochondrial enzyme in living cells, succinate-dehydrogenase, cleaves the tetrazolium ring, converting the MTT to an insoluble purple formazan. Consequently, the amount of formazan produced is directly proportional to the number of viable cells [21]. Extractions of *Actinidia* significantly inhibited cell growth both in HT-29 and HepG2 cells in a dose-dependent manner when compared to the control group (*p* < 0.05 or *p* < 0.01) (Figure 6a,b). Antiproliferative activity was expressed as the median effective dose (EC_50_) (Figure 6c). The extract of *Actinidia arguta* had a lower EC_50_ value in HepG2 and HT-29 cells compared to the *Actinidia kolomikta* or *Actinidia chinensis. Actinidia arguta* extract has the greatest antiproliferative activity, which was about 1.44–4.25-fold higher than the *Actinidia kolomikta* or *Actinidia chinensis*. This is likely due to the fact that *Actinidia arguta* extract has maximum flavonoid content. Therefore, *Actinidia arguta* extract has potential as a chemotherapeutic agent against HepG2 and HT-29 cells.

Previous studies have suggested a reverse relationship between the consumption of fruits and vegetables and the development of human chronic and degenerative diseases such as cancer [22–26]. This study found three *Actinidia* ethanol extracts had a high total phenolics and vitamin C content and showed significant potent radical scavenging activities in hydroxyl and O_2_^−^ radicals. The findings also showed that *Actinidia* extracts significantly inhibited the growth of HepG2 and HT-29 cells. Therefore, the total amount of both polyphenols and vitamin C were major contributors to the total antiproliferative capacity of *Actinidia* fruit.

Though many studies have shown an antiproliferative activity in phenolics extracts [27], the mechanisms of action are not yet clearly determined. Possible mechanisms include interference with the metabolite activation of promutagen, as blocking agents and formation of adducts with ultimate mutagens, scavenging of free radicals and suppression of tumor cell invasiveness via the inhibition of matrix metalloproteinase-2/-9 activity [28]. In this study, the positive effects of the antioxidants found in each *Actinidia* extract could contribute toward the antiproliferative activities of HT-29 and HepG2 tumor cells. Alternatively, taking into consideration the complexity of the mechanisms proposed for their chemopreventative properties, it is likely that anticarcinogenic effects attributed to polyphenols may be based on synergistic, additive, or antagonistic interactions of many compounds present in these extracts [29].

## 3. Materials and Methods

### 3.1. Chemicals and Reagents

The chemicals and reagents used in this study were as follows: ethanol, hydrochloric acid (37%), aluminum trichloride (AlCl_3_), 2,2-Diphenyl-picrylhydrazyl (DPPH), 2,2-Azino-bis(3-ethylbenzothiazoline- 6-sulfonic acid) diammonium salt (ABTS^+^), and ascorbic acid from Sigma Chemical Co. (St. Louis, MO, USA). Folin-ciocalteu phenol reagent, Gallic acid, Catechin, 30% hydrogen peroxide, salicylic acid, pyrogallol, and other chemicals were commercially provided from local suppliers (Harbin, China). All chemicals used in this study were of analytical reagent grade.

### 3.2. Sample Preparation

Three species of *Actinidia* (*Actinidia kolomikta*, *Actinidia arguta*, *Actinidia chinensis*) (Figure 1) were harvested from the Yi-Chun city (Heilongjiang Province, China). After which 200 g of each *Actinidia* was pulverized using a stainless steel blender and mixed with ethanol (60%) at the ratio of 1:5 (w/v). The mixtures were manually swirled for 30 min and placed in an ultrasonic apparatus (KX-1740QT) (50 °C, 100 W). The mixtures were centrifuged at 1500 × g for 5 min. Following this procedure, the residue was again ultrasonically extracted with ethanol (60%) under the same conditions and repeated twice. The supernatants were then individually pooled and condensed to 50 mL using a rotary evaporator. The final ethanol extracts from three *Actinidia* were maintained at −20 °C until further analysis. Each *Actinidia* species was extracted in triplicates.

### 3.3. Determination of Total Phenolic Content

The total phenolic content (TPC) of each *Actinidia* extract was determined by the Folin-Ciocalteu assay with a little modification [30,31]. This method adopted the following procedure: 100 μL of the standard gallic acid solution or appropriate dilutions of extraction was mixed with 1.9 mL of distilled water in a test tube followed by the addition of 1.0 mL Folin-Ciocalteu reagent. The samples were mixed well and then allowed to stand for 6 min; then 1.0 mL of aqueous sodium carbonate (100 g/L) was added. Samples were allowed to stand for 120 min at room temperature before the absorbance was measured at 765 nm versus the blank using a spectrophotometer (Shanghai, China). The results were expressed as mean (mg of gallic acid equivalents (GAE)/100 g fresh weight (FW) of sample) ± SD. Each sample was measured in triplicate.

### 3.4. Determination of the Total Flavonoid Content

The total flavonoid content (TFC) of each *Actinidia* extract was determined by the AlCl_3_ method with some modifications [1]. Briefly, 500 μL of the standard catechin solution or appropriate dilutions of extract was mixed with 2.5 mL of distilled water in a test tube, followed by the addition of 150 μL of 5% NaNO_2_ solution. Each sample was mixed well and then allowed to stand for 6 min, and then 300 μL of 10% AlCl_3_ solution was added. After the mixture reacted for 5 min, 1.0 mL of 1 mol/L of NaOH and 550 μL of distilled water was added. The absorbance values of each sample and the standard was measured at 510 nm versus the blank using a spectrophotometer (Shanghai, China). The results were expressed as mean (mg of catechin equivalents (CE)/100 g FW of sample) ± S.D. Each sample was measured in triplicate.

### 3.5. Determination of Vitamin C

The vitamin C content was assayed according to the Kampfenkel, Montagu, and Inze model [32]. Samples (2.0 g fruit) were homogenized in 8 mL of 6% (w/v) trichloroacetic acid pre-cooled on ice and centrifuged at 1000 × g for 20 min. The vitamin C content was determined based on the reduction of Fe^3+^ to Fe^2+^ by vitamin C in acidic solution. Fe^2+^ forms complexes with 2,2′-bipirydyl, to impart a pink color with a maximum absorbance at 525 nm. A standard curve of vitamin C was also used in this study. Each sample was measured in triplicate.

### 3.6. Hydroxyl Radical Scavenging Activity

The hydroxyl radical scavenging activity of each *Actinidia* ethanol extract was determined based on previous study [20]. The hydroxyl radical was generated through a Fenton reaction in the system of FeSO_4_ and H_2_O_2_. The reaction mixture consisted of 1.0 mL FeSO_4_ (9 mmol/L), 1.0 mL H_2_O_2_ (8.8 mmol/L), and 1.0 mL of various concentrations of each *Actinidia* extract and 1.0 mL of salicylic acid (9 mmol/L). The total mixture solution (4.0 mL) was incubated at 37 °C for 1 h and then the absorbance of the solution was recorded at 510 nm. The ascorbic acid was used as a positive control. The scavenging activity was calculated using Eqution (1). In this equation, A_0_ is the absorbance of the control (without extract), A_1_ is the absorbance of the extraction, and A_2_ is the absorbance without hydrogen peroxide. The regression of concentration and the scavenging rate were also analyzed in this study.

### 3.7. The O_2_^−^ Scavenging Assay

To examine O_2_^−^ scavenging capacity, 0.2 mL of different concentrations of each ethanol extract was placed into 5.7 mL of Tris-HCl buffer (50 mmol/L, pH 8.2) and incubated at room temperature for 5 min, then 0.1 mL of 6 mmol/L of pyrogallol (25 °C) was added. The absorbance of the reaction mixture was measured immediately at 320 nm and every 30 s until the reaction proceeded for 5 min (the same concentration of ethanol extract was used as the blank to eliminate interference). The O_2_^−^ scavenging activity was expressed by the oxidation degree of each group in comparison with that of the control group. The percentage of inhibition effect was calculated according to Equation (1), where A_0_ is the absorbance of the Tris-HCl buffer with pyrogallol, A_1_ is the absorbance of the extract and A_2_ is the absorbance of extract blank.

### 3.8. DPPH Radical Scavenging Activity Assay

The DPPH radical scavenging activity of each *Actinidia* extract was determined by following the method described by Reddy *et al.* [33,34]. In brief, the DPPH was dissolved in absolute methanol to a 0.2 mmol/L concentration; 2 mL of *Actinidia* extract of a different concentration (methanol is a dilute solution) was mixed with 2 mL of DPPH solution. The mixture was incubated at room temperature for 30 min. Finally, the absorbance of each mixture was measured using a spectrophotometer at 517 nm. The DPPH scavenging activity was calculated using Equation (1), where: A_0_, A_1_, and A_2_ are the absorbencies of the DPPH blank, sample, and sample blank, respectively.

### 3.9. The ABTS^+^ Method

The antioxidant capacity of the *Actinidia* ethanol extract was evaluated by colorimetric measurement using ABTS radical chromogens [35]. The antioxidant capacity of the extract to scavenge the ABTS radical cation (ABTS^+^) was generated by the reaction of 7 mmol/L aqueous solution of ABTS with a 2.45 mmol/L aqueous solution of K_2_S_2_O_8_ [36]. The solution was kept at room temperature for 16 h (in the dark) and diluted with water until the absorbance reached 0.7 ± 0.02 at 734 nm. Afterwards, 100 μL of different concentrations of ethanol extracts or ascorbic acid were mixed with 1.5 mL of an ABTS^+^ water solution, and placed at room temperature for 1 h (in the dark). The results were expressed as percentage of inhibition. All testing samples were repeated three times. The scavenging rate of antioxidant was calculated from Equation (1). Where A_0_ is the absorbance of the ABTS^+^ solution without adding sample, A_1_ is the absorbance of sample after acting with ABTS^+^ solution for 1 h, and A_2_ is the absorbance of the sample solution at 734 nm.

### 3.10. Determination of the Antiproliferative Activity

The antiproliferation activities on HepG2 and HT29 cell lines were examined using 3-(4,5-di-methylthiazol-2-yl)-2,5-diphenyl tetrazolium bromide (MTT) assay, as described in previous studies [28] with minor modifications. The HepG2 and HT29 cells were purchased from the Cancer Institute of the Chinese Academy of Medical Science (Beijing, China). The HepG2 cells were cultured in Dulbecco’s Modified Eagle Media (DMEM) and HT29 cells in RPMI 1640 with containing 10% fetal bovine serum (FBS) (Life Technologies, Gaithsburg, MD), 2 mmol/L L-glutamin, 100 U/mL of penicillin and 100 μg/mL of streptomycin. The cells were maintained at 37 °C in a humidified atmosphere of 5% CO_2_. HepG2 or HT29 cells were plated in a 96-well plate at 2.5 × 10^4^ cells/well. After 24 h, cells were treated with 100 μL of cell culture medium containing various concentrations (in triplicate) for 48 h at 37 °C. Ten microliters of 5.0 mg/mL of MTT solution in phosphate-buffered saline (PBS) was added to each well and incubated for 4 h. After careful removal of the medium, 150 μL of dimethyl sulfoxide (DMSO) was added to each well, and the plate was then shaken until the crystals were solubilized. The absorbance was recorded on a microplate reader (Elx800 Universal Microplate Reader, Bio-Tek Instruments) at a wavelength of 490 nm.

## 4. Conclusions

In summary, the *Actinidia* ethanol extracts were rich in phenolics, flavonoids and vitamin C. The *Actinidia* ethanol extracts also showed potent radical scavenging activities in hydroxyl and O_2_^−^ radicals and antiproliferative activities in HepG2 and HT-29 cells. This inhibition of cancer cell proliferation is at least partly due to the antioxidant activities of the extracts. Thus, *Actinidia* may be a potential source of natural antioxidants.

## Figures and Tables

**Table 1:** Total phenolic, total flavonoid and vitamin C contents of *Actinidia* extracts.

Variety of *Actinidia*	Total Phenolic Content (mg GAE/100 g FW)	Total Flavonoid Content (mg CE/100 g FW)	Vitamin C Content (mg ACE/100 g FW)
*Actinidia kolomikta*	430.03 ± 21.85	69.05 ± 0.75	211.12 ± 7.91
*Actinidia arguta*	362.18 ± 19.87	188.43 ± 3.65	26.97 ± 5.64
*Actinidia chinensis*	115.76 ± 8.97	67.63 ± 0.68	42.28 ± 0.77

**Table 2:** Correlation matrix showing the interrelation amongst phenol content, flavone content, vitamin C content, OH^−^, O_2_^−^ DPPH, ABTS scavenging. (-: signify repeat).

	Phenol	Flavone	Vitamin C	OH^−^	O_2_^−^	DPPH	ABTS
**Phenol**	1	-	-	-	-	-	-
**Flavone**	0.321	1	-	-	-	-	-
**Vitamin C**	0.609	−0.555	1	-	-	-	-
**OH**^−^	0.844	−0.236	0.939	1	-	-	-
**O_2_**^−^	0.712	−0.436	0.991	0.978	1	-	-
**DPPH**	0.787	−0.332	0.969	0.995	0.994	1	-
**ABTS**	0.975	0.105	0.769	0.941	0.849	0.903	1
